# Evaluation of the EQ-5D-3L and 5L versions in low back pain patients

**DOI:** 10.1186/s12955-021-01792-y

**Published:** 2021-05-28

**Authors:** A. M. Garratt, H. Furunes, C. Hellum, T. Solberg, J. I. Brox, K. Storheim, L. G. Johnsen

**Affiliations:** 1 Division for Health Services, Norwegian Institute of Public Health, Oslo, Norway; 2 Department of Surgery, Innlandet Hospital Gjøvik, Gjøvik, Norway; 3 University of Oslo, Oslo, Norway; 4 Division of Orthopaedic Surgery, Oslo University Hospital, Oslo, Norway; 5 Department of Neurosurgery and The Norwegian Registry for Spine Surgery (NORspine), University Hospital of Northern Norway, Tromsø, Norway; 6 Institute for Clinical Medicine, The Arctic University of Norway, Tromsø, Norway; 7 Department for Physical Medicine and Rehabilitation, Oslo University Hospital, Oslo, Norway; 8 Research and Communication Unit for Musculoskeletal Health (FORMI), Oslo University Hospital, Oslo, Norway; 9 Department of Physiotherapy, Oslo Metropolitan University, Oslo, Norway; 10 Department of Neuromedicine, Faculty of Medicine, Norwegian University of Science and Technology, Trondheim, Norway; 11 Department of Orthopaedic Surgery, St. Olav’s University Hospital, Trondheim, Norway

**Keywords:** EQ-5D-3L, EQ-5D-5L, Low back pain, Patient reported outcome measures, PROMs, Quality of life, Validity, Psychometrics

## Abstract

**Background:**

The EuroQol EQ-5D is one of the most widely researched and applied patient-reported outcome measures worldwide. The original EQ-5D-3L and more recent EQ-5D-5L include three and five response categories respectively. Evidence from healthy and sick populations shows that the additional two response categories improve measurement properties but there has not been a concurrent comparison of the two versions in patients with low back pain (LBP).

**Methods:**

LBP patients taking part in a multicenter randomized controlled trial of lumbar total disc replacement and conservative treatment completed the EQ-5D-3L and 5L in an eight-year follow-up questionnaire. The 3L and 5L were assessed for aspects of data quality including missing data, floor and ceiling effects, response consistency, and based on a priori hypotheses, associations with the Oswestry Disability Index (ODI), Pain-Visual Analogue Scales and Hopkins Symptom Checklist (HSCL-25).

**Results:**

At the eight-year follow-up, 151 (87%) patients were available and 146 completed both the 3L and 5L. Levels of missing data were the same for the two versions. Compared to the EQ-5D-5L, the 3L had significantly higher floor (pain discomfort) and ceiling effects (mobility, self-care, pain/discomfort, anxiety/depression). For these patients the EQ-5D-5L described 73 health states compared to 28 for the 3L. Shannon’s indices showed the 5L outperformed the 3L in tests of classification efficiency. Correlations with the ODI, Pain-VAS and HSCL-25 were largely as hypothesized, the 5L having slightly higher correlations than the 3L.

**Conclusion:**

The EQ-5D assesses important aspect of health in LBP patients and the 5L improves upon the 3L in this respect. The EQ-5D-5L is recommended in preference to the 3L version, however, further testing in other back pain populations together with additional measurement properties, including responsiveness to change, is recommended.

*Trial registration*: retrospectively registered: https://clinicaltrials.gov/ct2/show/NCT01704677.

## Background

The EQ-5D is one of the most widely tested and applied patient-reported outcome measures (PROM) worldwide. It has been translated into over 170 languages and national scoring algorithms exist for over 20 countries [1, 2]. Widespread application includes clinical and health services research, economic evaluation based on cost per quality adjusted life years (QALY) [3] and more recently, national quality measurement. The latter includes the National Health Service’s Patient Reported Outcome Measures (PROMs) programme for England [2] and medical registers in Norway and Sweden where it is the most widely used PROM [4, 5]. The EQ-5D is one of the mostly widely used PROMS for patients with low back pain (LBP) across these applications [3–8].

The EQ-5D-3L version includes five dimensions, or important aspects of health (mobility, self-care, usual activities, pain/discomfort and anxiety/depression), with three levels (no problem, some problems, severe problems) [2]. It is thus highly acceptable to patients and feasible for application where a short-form general measure of health is required. With the aim of improving the precision and responsiveness to change, the charity which owns the EQ-5D, the EuroQol Foundation, has developed the EQ-5D-5L [9], which has five levels, corresponding to none, slight, moderate, severe and extreme problems. There is strong evidence to suggest that the 5L will supplant the 3L version and the Norwegian Registry for Spine Surgery started using the former in 2019.

Based on the findings of recent systematic reviews and an international panel of experts, the EQ-5D was recommended for LBP [10, 11], but evidence for measurement properties has been deemed insufficient for widely used generic PROMs, including both versions of the EQ-5D [12]. The EQ-5D-3L has undergone limited evaluation in Norwegian patients with LBP [3, 13, 14] but it was concluded that the instrument is reliable and has evidence for validity supporting its use in economic evaluation [3]. Just two Chinese studies have assessed the 5L version in LBP and it was concluded that it was appropriate and valid [15, 16].

Following a systematic review that included 25 reports of concurrent or health-to-head comparisons of the EQ-5D-3L and 5L in diverse illness and healthy populations, it was found that the 5L showed similar or better measurement properties [9]. Updated systematic searches of PubMed lend further support to this finding based on a further twelve concurrent evaluations of data quality and measurement properties including validity and responsiveness to change, again in diverse illness groups and the general population [17–30]. It is important that the 5L is further evaluated for measurement properties in LBP [12]. Concurrent evaluation alongside the 3L will inform the choice of which version is the most appropriate [12, 31].

The study reported here is the first to consider the concurrent measurement properties of the two versions of the EQ-5D, administered at long-term follow-up, to patients with severe LBP randomized to rehabilitation or surgery with disc prostheses [14, 32]. The two versions are evaluated according to recognized measurement criteria for PROMs including those that have been used in previous comparisons of the 3L and 5L [9, 17–30].

## Methods

### Data collection

The study is an 8-year follow-up of a randomized multicentre study conducted at five university hospitals across Norway [32]. The trial included 173 patients aged 25–55 years randomised to rehabilitation or lumbar total disc replacement. Written informed consent was obtained and the inclusion criteria have been described [33]. PROMs were administered before randomization, and at 6 weeks, 3 months, 6 months, 1 year, 2 years and 8 years following the trial intervention. At the eight years endpoint of the trial, patients received a postal questionnaire and returned it in a reply-paid envelope before their follow-up visit [32].

The study was approved by the Norwegian Regional Committee for Medical Research Ethics South East C (2011/2177), conducted in accordance with the Helsinki Declaration and the ICH-GCP guidelines.

### Outcomes and psychological instruments

The eight-page self-completed questionnaire included the EQ-5D-3L and 5L on pages four and eight respectively. Health states from both versions are transformed to a single index using a scoring algorithm derived from valuation tasks undertaken with general population samples. An algorithm is not yet available for Norway and hence, recommendations of the Norwegian Medicines Agency [34] were followed, including the use of the UK value set [35] and mapping [36]. Scores for the EQ-5D index range from − 0.59 to 1, where 1 is the best possible health state. Summated rating scale scores were also computed for both versions to provide further information on the contribution of the additional two 5L response categories, in the absence of the scoring algorithm. In addition to the five dimensions, the EuroQol VisualAnalogue Scale (EQ VAS), assesses self-rated health on a vertical VAS, with endpoints labelled “Best imaginable health state” (100) and “Worst imaginable health state” (0).

The questionnaire also included the Norwegian version 2.0 of the Oswestry Disability Index (ODI) which has ten items assessing pain and daily activities with item-specific six-point descriptive scales [37]. ODI scores range from 0 to 100, with a lower score indicating less pain and disability. The instrument has evidence for reliability and validity in Norwegian patients with back pain [38]. Pain was assessed using visual analogue scale (VAS) measure of LBP ranging from 0 (no pain) to 100 (worst pain imaginable) relating to the back/hips and legs/feet [32]. Psychological distress was assessed by the Hopkin’s Symptom Check List (HSCL-25), which has 25 items assessing anxiety and depression symptoms during the last week [39]. Items have a four-point scale from “not at all” to “to a large extent” and sum to a score from 0 to 4, where 4 is the most severe symptoms [39, 40]. The instrument has been widely used in back pain research in Norway [8] and is considered acceptable for screening for depression in the Norwegian general population [40].

### Statistical analysis

Missing data and floor and ceiling effects were assessed for both versions of the EQ-5D. Following published comparisons of the 3L and 5L versions, classification efficiency was assessed using Shannon’s indices of H′, which assesses the extent to which information is evenly distributed across response categories, and J′, which also accounts for the number of response categories [9].

$$H^{\prime } = - \sum\limits_{{i = 1}}^{R} {p_{i} \ln p_{i} }$$

$$J^{\prime } = H^{\prime } {\text{/}}H_{{\max }} ^{\prime }$$

H′ can range from 0 to 1.58 for the 3L and 2.32 for the 5L, higher values indicating greater efficiency. J′ can range from 0 to 1, where 1 is greater efficiency with responses evenly distributed across categories [9].

Criteria for expected correlations between the EQ-5D and other instrument or item scores followed those included in a systematic review [12]. It has been argued that the EQ VAS is conceptually distinct to the EQ-5D index [41] but they both assess health in general, the latter including values for the health states assessed. Furthermore, if the five EQ-5D dimension scores make important contributions to health, then they should be highly correlated with the EQ-VAS scores. Hence, high levels of correlation ≥ 0.60 were expected between all aspects of the EQ-5D and the EQ VAS. However, slightly higher correlations were expected for the simple sum score of responses to the EQ-5D dimensions, because this does not include values for health states.

Whilst the EQ-5D is generic and the ODI relates to back pain, there is substantial overlap in content and scores for generic PROMs correlate moderate to highly with those specific to LBP [3, 15, 38]. Three ODI items (pain intensity, personal hygiene, walking) assess the same or very similar constructs as the EQ-5D, three assess aspects of usual activities (social life, sexual activity, travelling) and except for sleeping, there is considerable overlap for the remainder (lifting, sitting, standing). Except for anxiety/depression, high levels of correlation ≥ 0.60 were for expected between EQ-5D scores, and those for the ODI. Similar levels of correlation were expected between the EQ-5D pain/discomfort dimension and Pain-VAS scores. The HSCL-25 assesses anxiety and depression and except for the corresponding EQ-5D dimension, for which a high level of correlation ≥ 0.60 was expected, has very little overlap with the EQ-5D.

Correlations < 0.60 and ≥ 0.30 were expected for scores assessing largely related but dissimilar constructs: EQ-5D (index, sum, mobility, self-care, usual activities) and Pain-VAS; EQ-5D index, sum and HSCL-25. Correlations of < 0.50 and ≥ 0.20 were expected for scores assessing moderately related but dissimilar constructs: EQ-5D dimensions (except anxiety/depression) and HSCL-25; EQ-5D anxiety/depression and ODI. Finally, correlations < 0.30 were expected for instrument scores assessing weakly related or unrelated constructs: EQ-5D anxiety/depression with Pain-VAS scores. Furthermore, given the performance of the 5L relative to the 3L in other populations [9, 17–30], it was hypothesized that compared to those for the 3L, the 5L scores would have slightly higher correlations.

The five EQ-5D-3L and 5L dimension scores were compared with ODI categories of severity [16, 37] and anxiety/depression scores compared with the HSCL-25 cut-off point for diagnosis of psychiatric morbidity [40], by means of contingency tables and Chi-squared test. Finally, receiver operating characteristic curve analysis [42] was used to assess the discriminative ability of the EQ-5D-3L and 5L in discriminating between respondents with minimal versus moderate or worse disability for the ODI scores [16, 37]. The area under the curve ranges from 0.5 (no discriminative ability) to 1.0 (perfect discriminative ability).

Statistical analyses were undertaken using SPSS version 22.0 (IBM SPSS Statistics for Windows, IBM Corp., Armonk, NY).

## Results

### Data collection

There were 151 (87%) patients available for the 8-year follow-up. Table 1 shows their background characteristics and ODI and Pain-VAS scores. Their mean age was 50 (SD = 7.0) years and 53% were female.

**Table 1:** Patient characteristics at eight-year follow-up for those completing the EQ-5D-3L and 5L (n = 146)

	N	% (SD)
Mean age (years)	49.9	(7.0)
Female (%)	78	53.4
Work status:		
Working	61	41.8
Sick leave (100%)	10	6.9
Student	1	0.7
Sick leave (< 100%)	4	2.7
Pension	1	0.7
In rehabilitation	18	12.3
Disability pension	50	34.2
Unemployed	1	0.7
Mean ODI 8-year follow-up	24.75	(16.11)
ODI minimal disability (0–20)	61	41.8
ODI moderate disability (21–40)	62	42.5
ODI severe disability (41–60)	21	14.4
ODI crippling back pain (60–80)	2	1.4
ODI bed-bound (80–100)	0	0
Mean VAS-pain 8-year follow-up	42.10	(26.67)

### Statistical analysis

Missing data for both the 3L and 5L ranged from 1 to 2 dimensions and the same number of index scores were calculable; 146 patients completed both versions of the EQ-5D and are included in the results that follow.

For both versions of the EQ-5D and except for pain/discomfort, most patients reported none or slight/some problems across the five dimensions (Table 2). Apart from the self-care dimension and extreme problems, there were responses to all 3L response categories. For the 5L version, four and five of the response categories were used for three and two dimensions respectively. Floor effects (extreme problems), were very low for all but the pain/discomfort dimension where, compared to the 5L, 16% more patients had the worst level of pain for the 3L version. This was statistically significant. Ceiling effects (no problems), ranged from 18–73% and from 13–69% for the 3L and 5L respectively. Differences were statistically significant for all but the usual activities dimension. The largest difference between the two versions was for the mobility dimension, where a further 10% of patients reported no problems for the 3L. Based on response combinations to the five dimensions, the 146 patients had 28 (of a possible 243) and 73 (of a possible 3,125) separate health states as assessed by the 3L and 5L respectively. The mean (SD) index scores were 0.61 (0.32) and 0.66 (0.24) for the 3L and 5L respectively. Ceiling effects were 20 (14%) and 15 (11%) for the 3L and 5L respectively and the difference was not statistically significant. There were no floor effects.

**Table 2:** Response frequencies (%) for the EQ-5D-3L and 5L (n = 146)

EQ-5D-3L	No problems	Some problems	Extreme problems/unable to do
Mobility	92 (63.0)	52 (35.6)	2 (1.4)
Self-care	107 (73.3)	39 (26.7)	0 (0.0)
Usual activities	48 (32.9)	92 (63.0)	6 (4.1)
Pain/discomfort	26 (17.8)	92 (63.0)	28 (19.2)
Anxiety/depression	94 (64.4)	50 (34.2)	2 (1.4)

EQ-5D-5L	No problems	Slight problems	Moderate problems	Severe problems	Extreme problems/unable to do
Mobility	77 (52.7)**	42 (28.8)	21 (14.4)	6 (4.1)	0 (0.0)
Self-care	100 (68.5)*	37 (25.3)	5 (3.4)	4 (2.7)	0 (0.0)
Usual activities	46 (31.5)	68 (46.6)	19 (13.0)	8 (5.5)	5 (3.4)
Pain/discomfort	19 (13.0)*	52 (35.6)	43 (29.5)	28 (19.2)	4 (2.7)**
Anxiety/depression	85 (58.2)**	45 (30.8)	14 (9.6)	2 (1.4)	0 (0.0)

Asterisks denote statistically significant differences for McNemar’s related-samples change test: *P < 0.05; **P < 0.01

Table 3 shows response consistency. The great majority of patients reporting no problems for the 3L also report no problems for the 5L dimensions; 7–27% respond with slight problems on the 5L, the largest shift being for the pain/discomfort. The majority of patients reporting some problems for the 3L, report slight problems on the 5L; the pain dimension has a similar level of responses for both slight and moderate problems. Apart from the pain dimension, very few patients report extreme problems on the 3L, and the majority report severe rather than extreme problems on the 5L. Usual activities is the exception, where five of the six patients report extreme problems on the 5L. Between one and seven patients have response inconsistencies for each dimension, the highest being for the usual activities dimension, where six patients reporting some problems for the 3L report no problems for the 5L.

**Table 3:** Response consistency (%) between the EQ-5D-5L and EQ-5D-3L (n = 146)

EQ-5D-3L	EQ-5D-5L				
	No problems	Slight problems	Moderate problems	Severe problems	Unable/extreme
Mobility					
No problems (n = 92)	77 (83.7)	14 (15.2)	**1 (1.1)**	**0 (0.0)**	**0 (0.0)**
Some problems (n = 52)	**0 (0.0)**	28 (53.8)	20 (38.5)	4 (7.7)	**0 (0.0)**
Unable/extreme (n = 2)	**0 (0.0)**	**0 (0.0)**	**0 (0.0)**	2 (100.0)	0 (0.0)
Self-care					
No problems (n = 107)	99 (92.5)	8 (7.5)	**0 (0.0)**	**0 (0.0)**	**0 (0.0)**
Some problems (n = 39)	**1 (2.6)**	29 (74.4)	5 (12.8)	4 (10.3)	**0 (0.0)**
Unable/extreme(n = 0)	**0 (0.0)**	**0 (0.0)**	**0 (0.0)**	0 (0.0)	0 (0.0)
Usual activities					
No problems (n = 48)	40 (83.3)	8 (16.7)	**0 (0.0)**	**0 (0.0)**	**0 (0.0)**
Some problems (n = 92)	**6 (6.5)**	60 (65.2)	18 (19.6)	8 (8.7)	**0 (0.0)**
Unable/extreme (n = 6)	**0 (0.0)**	**0 (0.0)**	**1 (16.7)**	0 (0.0)	5 (83.3)
Pain/discomfort					
No problems (n = 26)	19 (73.1)	7 (26.9)	**0 (0.0)**	**0 (0.0)**	**0 (0.0)**
Some problems (n = 92)	**0 (0.0)**	45 (48.9)	41 (44.6)	6 (6.5)	**0 (0.0)**
Unable/extreme (n = 28)	**0 (0.0)**	**0 (0.0)**	**2 (7.1)**	22 (78.6)	4 (14.3)
Anxiety/depression					
No problems (n = 94)	85 (90.4)	9 (9.6)	**0 (0.0)**	**0 (0.0)**	**0 (0.0)**
Some problems (n = 50)	**0 (0.0)**	36 (72.0)	13 (26.0)	1 (2.0)	**0 (0.0)**
Unable/extreme (n = 2)	**0 (0.0)**	**0 (0.0)**	**1 (50.0)**	1 (50.0)	0 (0.0)

Inconsistencies are marked in bold

Shannon’s H’ ranged from 0.25 (self-care) to 0.40 (pain/discomfort) and from 0.36 (self-care) to 0.61 (pain/discomfort) for the 3L and 5L items respectively. J′ ranged from 0.12 (self-care) to 0.28 (pain/discomfort) and from 0.17 (self-care) to 0.47 (pain/discomfort) for the 3L and 5L items respectively. Compared to the 3L, 5L dimensions showed mean information gain ranging from 1.36 (anxiety/depression) to 1.60 (usual activities) for H′ and from 1.38 (anxiety/depression) to 1.70 (pain/discomfort) for J′.

Table 4 shows the correlations between the EQ-5D-3L and 5L scores and those for the other instruments. Compared to the 3L, 5L dimension, sum and index scores had slightly higher correlations with those for the EQ VAS. For both versions, the highest correlations with ODI scores were found for the pain/discomfort dimension followed by correlations ≥ 0.60 for all but anxiety/depression. The pain/discomfort dimension had the highest correlations of 0.66–0.82, with the two Pain-VAS scores. Apart from anxiety/depression, correlations with Pain-VAS scores were higher than expected. Correlations with the HSCL-25 were ≥ 0.60 for anxiety/depression, otherwise mostly < 0.50 for other dimensions. The two index scores had correlations ≥ 0.60 with those for other instruments. The EQ-5D-5L had slightly higher correlations than the 3L with other instrument scores; the largest of up to 0.10 were for the pain/discomfort dimension. Compared to the index scores, EQ-5D sum scores had slightly higher correlations with those for the other instruments, except for the two Pain-VAS scales and the 5L.

**Table 4:** Listwise Spearman correlations^a^ (n = 146)

	EQ VAS	Oswestry Disability Index	Pain-VAS Back/Hips	Pain-VAS Legs/feet	HSCL-25
EQ-5D-3L index	0.72	0.84	0.76	0.64	0.65
EQ-5D-3L sum^b^	0.76	0.87	0.77	0.66	0.66
Mobility	0.57	0.68	0.57	0.61	0.41
Self-care	0.56	0.65	0.59	0.46	0.37
Usual activities	0.65	0.72	0.65	0.49	0.51
Pain/discomfort	0.62	0.77	0.75	0.66	0.45
Anxiety/depression	0.39	0.36	0.26	0.19NS	0.64
EQ-5D-5L index	0.74	0.87	0.82	0.70	0.68
EQ-5D-5L sum	0.77	0.88	0.80	0.69	0.69
Mobility	0.59	0.72	0.62	0.60	0.45
Self-care	0.56	0.66	0.62	0.48	0.46
Usual activities	0.73	0.75	0.68	0.57	0.54
Pain/discomfort	0.68	0.85	0.82	0.76	0.51
Anxiety/depression	0.40	0.41	0.31	0.17NS	0.72

^a^All correlations are statistically significant (P < 0.01) unless indicated: NS not significant. Scores are coded in the same direction to ease interpretation

^b^EQ-5D-3L and 5L index are based on recommended scoring, whereas the sum scores are the sum of the scores for the five dimensions

Table 5 shows responses to the EQ-5D dimensions by ODI categories of severity and anxiety/depression by HSCL-25 cut-off for psychiatric diagnosis. Compared to the 3L, more response categories were used for the 5L across ODI severity levels and particularly for moderate and severe levels. For both versions, there was the same number of respondents below the HSCL-25 cut-off. For those at or above the cut-off, there was a greater spread of 5L responses compared to the 3L. Chi-squared values were consistently higher for the 5L. Both EQ-5D-3L and 5L index scores were statistically significant in discriminating between respondents with ODI scores indicating minimal and higher levels of severity. The area under the curve (95% CI) for the 3L and 5L was 0.946 (0.914–0.977) and 0.956 (0.928–0.984) respectively. The results were very similar for the EQ-5D sum scores.

**Table 5:** EQ-5D responses^a^ (%) for ODI and HSCL-25 severity levels (n = 146)

		ODI classification			
		Minimal	Moderate	Severe/crippling	Chi-square^b^
EQ-5D-3L					
Mobility	No problems	59 (40.4)	31 (21.2)	2 (1.4)	69.90
	Some problems	2 (1.4)	31 (21.2)	19 (13.0)	
	Unable/extreme	–	–	2 (1.4)	
Self-care	No problems	61 (41.8)	43 (29.5)	3 (2.1)	65.36
	Some problems	–	19 (13.0)	20 (13.7)	
Usual activities	No problems	43 (29.4)	5 (3.4)	–	71.14
	Some problems	18 (12.3)	54 (37.0)	20 (13.7)	
	Unable/extreme	–	3 (2.1)	3 (2.1)	
Pain/discomfort	No problems	26 (17.8)	–	–	92.85
	Some problems	35 (24.0)	51 (34.9)	6 (4.1)	
	Unable/extreme	–	11 (7.5)	17 (11.6)	
Anxiety/depression	No problems	50 (34.2)	37 (25.3)	7 (4.8)	22.42
	Some problems	10 (6.8)	25 (17.1)	15 (10.3)	
	Unable/extreme	1 (0.7)	–	1 (0.7)	
EQ-5D-5L					
Mobility	No problems	57 (39.0)	19 (13.0)	1 (0.7)	99.84
	Slight problems	3 (2.1)	32 (21.9)	7 (4.8)	
	Moderate problems	1 (0.7)	10 (6.8)	10 (6.8)	
	Severe problems	–	1 (0.7)	5 (3.4)	
Self-care	No problems	60 (41.1)	36 (24.7)	4 (2.7)	78.48
	Slight problems	1 (0.7)	25 (17.1)	11 (7.5)	
	Moderate problems	–	1 (0.7)	4 (2.7)	
	Severe problems	–	–	4 (2.7)	
Usual activities	No problems	41 (28.1)	5 (3.4)	–	85.43
	Slight problems	19 (13.0)	41 (28.1)	8 (5.5)	
	Moderate problems	1 (0.7)	10 (6.8)	8 (5.5)	
	Severe problems	–	3 (2.1)	5 (3.4)	
	Unable/extreme	–	3 (2.1)	2 (1.4)	
Pain/discomfort	No problems	19 (13.0)	–	–	125.34
	Slight problems	36 (24.7)	16 (11.0)	–	
	Moderate problems	6 (4.1)	33 (22.6)	4 (2.7)	
	Severe problems	–	13 (8.9)	15 (10.3)	
	Unable/extreme	–	–	4 (2.7)	
Anxiety/depression	No problems	48 (32.9)	31 (21.2)	6 (4.1)	27.75
	Slight problems	10 (6.8)	25 (17.1)	10 (6.8)	
	Moderate problems	2 (1.4)	6 (4.1)	6 (4.1)	
	Severe problems	1 (0.7)	–	1 (0.7)	
EQ-5D-3L		HSCL-25 < 1.75	HSCL-25 ≥ 1.75		
Anxiety/ depression	No problems	77 (54.6)	15 (10.6)		40.15
	Slight problems	15 (10.6)	33 (23.4)		
	Unable/extreme	–	1 (0.7)		
EQ-5D-5L					
Anxiety/ depression	No problems	72 (51.1)	11 (7.8)		51.74
	Slight problems	20 (14.2)	23 (16.3)		
	Moderate problems	–	13 (9.2)		
	Severe problems	–	2 (1.4)		

^a^Response categories not included where there were no responses

^b^All results are statistically significant: p < 0.001

## Discussion

The concurrent nature of this study represents the strongest available evidence for choosing the most recent version of the EQ-5D with five levels in LBP research and other forms of application. Levels of missing data were similar for both versions and low. Across EQ-5D-5L dimensions, patients used four or five response categories and hence described a greater range of health states than for the 3L (73 versus 28). There was a significantly higher floor effect for the pain/discomfort dimension for the 3L version, an important aspect of health in these patients. Ceiling effects were large across both instruments, which was expected in this long-term follow-up of patients [32]. There was little difference between the two versions for the usual activities dimension, but for the remaining four dimensions there were statistically significant differences in favour of the 5L.

One systematic review that included comparisons of the two versions in various illness groups and the general population [9], also found low levels of missing data for both versions, and that using the 5L could reduce ceiling effects by up to 17% for mobility and 30% for self-care dimensions. Floor effects were found to be largely below 5% across dimensions, but in common with the findings reported here, the largest reduction from using the 5L was found for pain/discomfort [9]. The review included patient populations that were not part of a long-term follow-up and hence larger differences in ceiling effects might be expected compared to the results reported here. More recent comparisons across diverse illness groups have found statistically significant reductions in ceiling effects for the 5L relative to the 3L [22, 24–26, 28], with the pain/discomfort dimension often being the largest and ranging from 5 to 17% for Crohn’s disease [22] and older people with moderate to high levels of comorbidity [25] respectively.

The assessment of response consistency was limited because very few patients scored at the floor or the poorest level of health for both versions. The few inconsistencies were magnified by the small samples available. Shannon’s indices also showed that the 5L outperformed the 3L in tests of classification efficiency which was found in previous studies [17, 18, 21–26, 29].

The inclusion of the ODI, Pain-VAS and HSCL-25 in correlations based on bypothesis testing, follows existing LBP studies [3, 8, 12, 13, 38], and hence, was important for assessing the comparative performance of the EQ-5D-3L and 5L. Compared to the 3L, the 5L index and dimension scores had higher levels of correlation with those for these instruments and were more highly associated with ODI and HSCL-25 levels of severity. The largest differences were for the pain/discomfort dimension, which reflects the content of the ODI, Pain-VAS items and their specific focus on LBP. Together, the findings show that the EQ-5D assesses important aspect of health in LBP, and that the 5L improves upon the 3L in this respect. The findings of a recent systematic review highlighted the need for further testing for the construct validity of the EQ-5D-5L in LBP patients [12]. Two Chinese studies have since concluded that the 5L has evidence for validity in these patients [15, 16]. Compared to the findings reported here, slightly lower levels of correlation with the ODI were reported in a sample of outpatients [15]. In a sample that also included in-patients, higher AUC scores were found for the EQ-5D-5L compared to the SF-6D, and in relation to the ODI severity categories reported here [16].

### Study strengths and limitations

The EQ-5D-5L was not available when the randomized trial began [32], which constrained the study design and measurement properties tested. Study strengths include the concurrent nature of the evaluation which gives the strongest available evidence for comparative measurement performance [9, 12, 31]. However, the ordering of two versions of the EQ-5D may have affected results. The study was part of an eight-year follow-up of a randomized trial which defined the questionnaire layout and ordering of the PROMs. Had the study been primarily concerned with comparing the EQ-5D-5L and EQ-5D-3L, then randomizing patients to two questionnaires, one with the 5L and one with the 3L, would have been the preferred design. This would also have alleviated any concerns that completing the 3L prior to the 5L might have influenced responses to the latter. The 3L came first because it was used at baseline, and hence was an important outcome measure within the trial. There is no way of testing for such potential biases within the current design. The questionnaire was brief with eight pages of A4 and hence there is limited grounds to expect that respondent burden may have contributed to the 5L version performing poorer relative to the 3L version.

The longitudinal nature of the main study also limited the measurement properties that could be tested and previously reported for the EQ-5D-3L in LBP patients, including reliability and responsiveness to change [3, 12, 14]. Furthermore, the design precluded estimating the standard error of measurement and minimal detectable change. It is recommended that these measurement criteria are considered in future testing of the EQ-5D-5L in LBP patient populations. The current findings, together with those from other studies that included LBP patients [15, 16] and other populations [9, 17–30], indicate that the results of further testing for measurement properties including responsiveness to change, will favour the 5L.

There is currently no Norwegian value set or scoring algorithm for the EQ-5D-5L. Norwegian data was being collected for this purpose [43] but was postponed because of the COVID-19 pandemic. In the absence of a Norwegian scoring algorithm, scoring of the EQ-5D-3L and 5L index followed existing recommendations [34]. The analyses undertaken here should be replicated for the EQ-5D-5L index when a Norwegian EQ-5D value set and scoring algorithm become available. Norwegian medical registers including the National Register for Spine Surgery [4], recently supplanted the 3L with the 5L, and the findings here support the national recommendations [34] that they follow.

## Conclusions

The EQ-5D is the most widely used short generic instrument suitable for use in economic evaluation including cost per QALY calculations. These results support the use of the 5L in preference to the 3L version but further and more extensive testing in other LBP populations is recommended.

## Data Availability

Data supporting our conclusions can be found at the Communication- and Research Unit for Musculoskeletal Disorders (FORMI), Oslo University Hospital & University of Oslo, Ullevaal, P.O. Box 4950, Nydalen, 0424 Oslo.

## Abbreviations

EQ-5D: EuroQol EQ-5D
HSCL-25: Hopkin’s symptom check list
LBP: Low back pain
ODI: Oswestry Disability Index
Pain-VAS: Pain Visual Analogue Scale
PROMs: Patient-reported outcomes measures
SD: Standard deviation
